# Plasma GFAP and *p*‐tau 181 were closely linked with longitudinal synaptic loss and cognition decline

**DOI:** 10.1002/alz70856_101566

**Published:** 2025-12-25

**Authors:** Jie Wang

**Affiliations:** ^1^ Huashan Hospital, Fudan University, Shanghai, Shanghai, China

## Abstract

**Background:**

To investigate the associations of longitudinal synaptic loss and cognition decline with tau pathology and neuroinflammation measured by positron emission tomography (PET) and plasma analysis in Alzheimer's disease (AD).

**Method:**

Twenty cognitively impaired individuals (CI) and sixteen healthy controls (HC) underwent cognitive and plasma biomarker assessments, amyloid‐PET ([18F]Florbetapir), tau‐PET ([18F]MK6240), and synaptic density PET ([18F]SynVesT‐1), after one year, tau and synaptic density PET, and cognitive assessments were repeated. Student's t‐test analyzed group differences in longitudinal synaptic loss and tau burden increase. The relationships among tau pathology, plasma biomarkers, synaptic density and cognition were investigated based on partial correlation analyses, general linear models and mediation analyses.

**Result:**

The CI group had more longitudinal synapse loss in the medial temporal lobe and tau burden increases in the widespread neocortex than the HC group. In the whole cohort, the longitudinal change of tau burden was negatively associated with the longitudinal change of synaptic density, and the baseline tau burden, plasma *p*‐tau 181, NfL and GFAP were negatively associated with synaptic density at the time of baseline and follow‐up, and the baseline tau burden, plasma *p*‐tau 181 and GFAP could predict the longitudinal change of synaptic density. Mediation analyses revealed that plasma GFAP mediates the relationship between baseline tau pathology and synaptic density both at the time of baseline and follow‐up, longitudinal change of tau burden and longitudinal change of synaptic density. The longitudinal change of synaptic density was positively associated with the longitudinal change of MMSE scores, the baseline synaptic density, plasma *p*‐tau 181 and GFAP could predict the longitudinal cognition decline, tau pathplogy mediates the relationship between baseline plasma GFAP and longitudinal cognition decline.

**Conclusion:**

CI patients have more longitudinal synapse loss and tau burden increases with one‐year follow‐up than HC participants. Baseline tau pathology(tau burden and plasma *p*‐tau 181) and plasma GFAP were negatively associated with synaptic density and could predict longitudinal synapse loss. Tau pathology might trigger neuroinflammation leading to synaptic loss. Plasma *p*‐tau 181 and GFAP could predict the longitudinal cognition decline. Tau pathology and neuroinflammation interaction resulted in synaptic loss and cognition decline.

